# The moderating effect of lifetime physical activity on brain alterations related to adverse childhood experiences

**DOI:** 10.1192/j.eurpsy.2025.10116

**Published:** 2025-10-20

**Authors:** Lemye Zehirlioglu, Traute Demirakca, Richard Nkrumah, Lennart Ettingshausen, Yasmin Grauduszus, Claudius von Schröder, Melissa Feichtmair, Nikolaus Kleindienst, Gabriele Ende, Christian Schmahl

**Affiliations:** 1 Department of Psychosomatic Medicine and Psychotherapy, Central Institute of Mental Health, Medical Faculty Mannheim, Heidelberg University, Mannheim, Germany; 2 German Center for Mental Health (DZPG), Partner Site Mannheim – Heidelberg, Ulm, Germany; 3 Department of Neuroimaging, Central Institute of Mental Health, Medical Faculty Mannheim, University of Heidelberg, Mannheim, Germany

**Keywords:** adverse childhood experiences, amygdala, brain volume, hippocampus, limbic system, physical activity

## Abstract

**Background:**

Adverse childhood experiences (ACEs) can cause morphological brain alterations across the lifespan, contributing to increased vulnerability to mental and physical disorders. Despite extensive research on ACEs-related brain alterations, the protective or augmenting role of modifiable lifestyle factors such as physical activity has been largely underexplored, representing a key gap in our understanding of trauma-related neuroplasticity. To close this gap, we aimed to investigate how lifetime physical activity (LPA) influences the relationship between ACEs and morphological brain alterations.

**Methods:**

Moderation analyses using Hayes’ PROCESS macro examined the interaction between ACEs and LPA on the volume of limbic system-related regions – hippocampus, amygdala, anterior cingulate cortex (*n* = 81).

**Results:**

While LPA showed no moderating effect on hippocampal or anterior cingulate volume, the model concerning the volume of the amygdala was significant. This model explained 8.1% of the variance in amygdala volume (*p* = 0.002) and the interaction of LPA and ACEs contributed 7.9% of this variance, with a significant effect (*β* = −0.221 *p* ≤ 0.001). That indicated LPA moderates ACEs-related structural changes in the amygdala, a key component of the central circuitry of emotion and stress sensitization. Notably, only in individuals with low physical activity were ACEs associated with increased volume of amygdala.

**Conclusions:**

Our findings underscore the behavioral dependency of the structural adaptations of the amygdala following childhood adversities. These results emphasize the therapeutic potential of incorporating physical activity into interventions for trauma-exposed individuals, offering a behavioral approach to mitigating stress-related neurobiological changes.

## Introduction

Adverse childhood experiences (ACEs), such as abuse (emotional, physical, and sexual) and neglect (emotional and physical), have far-reaching, long-lasting negative effects that can manifest even years later in adulthood [1–3]. The critical importance of ACEs has been emphasized due to its increasing prevalence and mental and physical consequences including depression, anxiety [4], posttraumatic stress disorder (PTSD) [5], borderline personality disorder (BPD) [6], obesity [7], and cardio-metabolic diseases [2]. While it is clear that individuals exposed to ACEs have a higher risk of developing such disorders than those who are not exposed, this association is thought to involve disruptions in neurobiological development. One important factor is increased activity in the hypothalamic–pituitary–adrenal axis (HPA) due to chronic stress, resulting in altered brain structure and function in adulthood [2, 3, 8]. The amygdala, hippocampus, and anterior cingulate cortex (ACC) are the most frequently investigated brain regions in studies on ACEs. These regions are central to the brain’s stress response system and are densely interconnected with the HPA axis. The amygdala is critical for salience detection and emotional reactivity and is highly sensitive to glucocorticoid exposure [9]. The hippocampus supports contextual memory and regulates negative feedback within the HPA axis, making it particularly vulnerable to chronic stress [10]. The ACC is involved in emotion regulation, attention, and conflict monitoring, and plays a modulatory role across both limbic and cognitive networks [11, 12]. Structurally the hippocampus and ACC have been commonly found to be smaller in ACEs affected subjects [13, 14], although some studies, contrary to the general trend, have failed to find any alterations [15–18]. Findings on the amygdala volume have been more conflicting, with studies reporting both increases and decreases in volume [19, 20]. Although there is broad consensus about the vulnerability of these regions to stress, inconsistencies shows that further investigation is needed to clarify the mechanisms underlying these associations. Exploring how behavioral factors such as physical activity (PA) interact with these regions might provide critical insights.

PA has been known as a preventive measure as well as a powerful, cost-effective, side-effects-free treatment for both physical and mental health problems [21, 22]. Various studies have shown that prescribing PA is effective in improving health outcomes in mental disorders [23]- such as schizophrenia, bipolar disorder [24], depression [25] anxiety [26] and post-traumatic stress disorder (PTSD) [27] - and helps to reduce residual symptoms, poor quality of life, poor and enhance treatment adherence [28]. PA may promote brain health by stimulating the release of neurotrophic factors such as brain-derived neurotrophic factor (BDNF), which supports emotion regulation and boosts neuronal growth and plasticity [29]. Neuroimaging studies have shown that PA is highly associated with morphological changes in stress-sensitive brain regions [30]. These changes include increased volumes of the hippocampus [22, 31], and the ACC [12, 22], as well as decreased amygdala volume [32].

The current PA literature in ACEs samples has mainly focused on the influence of ACEs on PA levels, with the majority indicating that PA levels are low among individuals with ACEs. Few studies have focused on the impact of PA on mental and/or physical health among adults with history of ACEs [33–35]. The possible effect of PA on ACEs-related brain alterations was reviewed by Donofry et al. (2021). They stressed the absence of research on this topic and recommended that cross-sectional studies should be conducted as an initial step to understand the relationship between physical activity and brain health among people with a history of ACEs [30].

To close this gap, we aimed to investigate how lifetime physical activity (LPA) influences the relationship between ACEs and morphological brain alterations. We hypothesized that ACEs’ effect on brain volume is moderated by LPA. To test this, we created moderation models for three region of interest: (1) hippocampus (2), amygdala, and (3) ACC. These three ROIs were selected based on converging evidence that they are sensitive to ACE exposure and responsive to PA-induced neuroplasticity, as outlined above, making them strong candidates for testing brain-based resilience mechanisms. Given that the existing literature typically assesses physical activity over relatively short periods – such as a week or a year – yet evidence suggests that cumulative PA levels have the most significant impact on brain health in healthy populations [36], our study specifically focuses on LPA. By this approach, we aim to underscore the importance of physically active lifestyle among this population. Findings from this research may not only contribute to a deeper understanding of the mechanisms underlying ACEs-related brain alterations but also might shape preventive interventions brain health and resilience in at-risk populations.

## Materials and methods

### Design and sample

Data for this study were collected as part of the Research Training Group “The impact of ACEs on psychosocial and somatic conditions across the lifespan,” (GRK2350) funded by the German Research Foundation. Between January 2019 and June 2024, a total of 169 adult participants were recruited into the GRK2350 ‘Brain Morphology’ study group (https://osf.io/s5ydb/), primarily through outpatient clinic referrals, distributed flyers, and community advertisements. Trauma history was assessed using a battery of psychometric instruments, including the Childhood Trauma Questionnaire (CTQ) [37]. Individuals aged between 18 and 60 years were eligible to participate if they reported any type of abuse (physical, emotional, or sexual) and/or neglect (emotional or physical) prior to the age of 18. Exclusion criteria included standard MRI contraindications (e.g., metal implants, pregnancy). Although psychiatric diagnoses were not part of the recruitment criteria, additional exclusion criteria were applied to ensure data quality and safety. These included lifetime diagnoses of psychotic or bipolar-I disorders, current substance use disorders, and recent use of psychotropic medication, except for stable antidepressant treatment. The recruitment procedures and full exclusion criteria have been described in detail in earlier publications from our group [13, 14, 38]. All participants with available CTQ and MRI data who had given permission to be re-contacted were considered eligible for this follow-up study (*N* = 128). These individuals were invited to complete an online survey that included the Lifetime Leisure Physical Activity Questionnaire (LLPAQ) [39]. As compensation, participants were entered into a lottery for several €30 vouchers. The survey return rate was 69%; after excluding responses with missing or implausible data (e.g., unrealistically high values or non-numeric entries), the final sample comprised 81 participants (83,3% female) (Table 1). Based on structured clinical interviews and validated self-report instruments, 11.1% of the final sample did not meet criteria for any lifetime psychiatric disorder, while 55.6% screened positive for Posttraumatic Stress Disorder (PTSD) according to the Posttraumatic Stress Disorder Checklist (PCL) [40].

**Table 1.** Description of the sample (*n* = 81)

	Median (SD)	Min–max	Correlations		
			Age	CTQ	Min_A_
Age	29.00 (10.56)	18–58			
CTQ	63.00 (20.88)	26–106	.261*		
Min_A_	239.42 (275.90)	15–1324	−0.062	−0.149	

*Note:* Significant correlations are indicated in bold; *p < 0.05.

Abbreviations: CTQ, Childhood trauma questionnaire; Min_A_, lifetime average weekly physical activity minutes; SD, standard deviations.

## Measures and procedure

### Adverse childhood experiences

The CTQ was developed as a retrospective screening tool for histories of childhood maltreatment and refer to the first 18 years of participants’ lives [41]. Studies demonstrating the validity of the measure use psychometric evaluations of the CTQ and the subscale scores in a variety of populations, psychiatric samples, and community samples [37]. The questionnaire consists of self-reported 28 items covering five different types of maltreatment – emotional, physical, and sexual abuse, as well as emotional and physical neglect. Subjects indicated the occurrence of maltreatment on 5-point Likert scales (1 = never true, 5 = very often true). The score can be interpreted for each subscale (from 5 = no maltreatment to 25 = extreme maltreatment) and as a global severity index across all subscales. Here, we calculated the overall CTQ total score as the sum of all subscales (min: 25, max: 125).

### Lifetime physical activity (LPA)

LPA data was collected via the LLPAQ [39, 42]. The LLPAQ assesses the history of 24 different leisure time physical activities. Respondents who had performed an activity at least 10 times in their lifespan were asked to provide detailed information. The questionnaire collects lifetime activity in epochs of 15 years, only the first 20 years of life are captured together (0–20 years, 21–35 years, 36–50 years, 51–65 years). Within every epoch, the number of activities is assessed with questions regarding hours per week (length), months per year (frequency) and years per episode (duration).

The hours from all epochs and from each activity were summed up for the Total Lifetime Hours. To get a more comparable measure between subjects, independent of age, finally the Total Lifetime Hours were divided by age and 52 (1 Year = 52 Weeks) to get hours per week. This value was then multiplied by 60 (1 h = 60 min) to get an average of “minutes per week.” By that equation (1) total lifetime hours were converted to *Lifetime Physical Activity Average Weekly Minutes (Min_A_).*


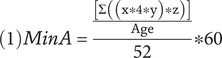



Min_A_ = lifetime average physical activity weekly minutes.

*x* = hours per week (length) *y* = months per year (frequency) *z* = years per episode (duration).

The psychometric properties of the LLPAQ were assessed in a sample of adults aged 75–90 years, demonstrating strong reliability in evaluating long-term physical activity patterns. The test–retest reliability of the LLPAQ was excellent (*r* = 0.824, *p* < 0.001), and internal consistency yielded a Cronbach’s alpha of 0.80 (*p* < 0.001). While the questionnaire showed a significant correlation (*r* = 0.311, *p* = 0.033) between accelerometer-measured physical activity over a one-week period and self-reported physical activity over the previous year, the LLPAQ was selected for use in this study based on its high test–retest reliability, which underscores its robustness in capturing cumulative physical activity exposure throughout lifespan [39].

### Magnetic resonance imaging and image processing

Magnetic resonance imaging was conducted on a Siemens 3 Tesla MAGNETOM Prisma^fit^ (Siemens Medical Solutions, Erlangen Germany) and images were acquired with a 64-channel head coil (MPRAGE; T1-weighted contrast, Echo Time (TE): 2.01 ms; Repetition Time (TR): 2000 ms; Inversion time (TI): 900 ms; FA = 9°; FOV: 256 x 256 mm; number of slices 192, voxel size 1x1x1 mm^3^). Anatomical data were preprocessed and analyzed with the Computational Anatomy Toolbox (CAT12; http://www.neuro.uni-jena.de/cat), based on SPM12 (Welcome Department of Imaging Neuroscience, London, UK) running on MATLAB (2022a. Natick, Massachusetts: The MathWorks Inc, USA). After quality control, one subject was excluded. Native anatomical images were segmented into gray and white matter and CSF. The gray matter images were normalized into MNI –space and smoothed with a 12 mm full width at half maximum (FWHM) isotropic Gaussian kernel. Gray matter volume of the ROIs was estimated using CAT 12 on the basis of the Neuromorphometic atlas (provided by Neuromorphometrics, Inc., MA, USA; http://www.neuromorphometrics.com). The volume of left and right hemispheres summed and total volume of ROIs were used for analysis. ROIs were the amygdala, hippocampus, anterior cingulate gyrus (ACG). The unit for the volume is ml and all ROI volumes were adjusted for estimated Total Intracranial Volume (eTIV), provided by CAT12. All analyses were conducted on the residuals of the ROI volumes after adjusting for age and sex, ensuring that these variables were incorporated into the statistical models.

## Data analysis

The main hypothesis – moderation effect of LPA on the relationship between ACEs and ROIs volume [(1) hippocampus (2) amygdala and (3) ACC)] – was tested with SPSS 26 and PROCESS Macro extension by Hayes [43]. The PROCESS is an add-on tool developed based on logistic regression path analysis and allows to investigate conditional effects. The ‘conditional effect’ term refers to a setting in which the relationship between two variables is not constant and is dependent upon the values of a third variable, which is referred to as a moderator variable. The relationship between moderator and the independent variable is defined as the interaction term and the significance of interaction term indicates that the strength or direction of the relationship between the independent and dependent variables varies depending on the level of the moderator, thereby confirming the presence of conditional effect and supporting significant moderation model [43].

To ensure data accuracy, responses to the LLPAQ were carefully screened, the reported weekly hours per activity were evaluated for plausibility. In cases where responses indicated more than 20 hours per week for a single activity, value was capped at 20 hours. This threshold was based on reasonable expectations for sustained leisure physical activity and aimed to minimize the impact of outliers on the dataset. Preliminary analysis indicated that the LPA data was positively skewed. This distribution thought to be consistent with population-based observations of leisure time physical activity. Variables were z-transformed by subtracting the mean of the variable and then dividing by the standard deviation to ensure both the moderator and independent variable were scaled to unit variance. To assess whether the relationships between variables met the linearity assumption, visual inspection of the scatterplots supported by locally weighted linear regression (LOESS) was applied for all variables included in the moderation analysis [44]. The LOESS curve closely aligned with fitted linear regression line, indicating that the relationships were well-fitted and the linearity assumption was met. To ensure no violation of the homoscedasticity assumption, heteroscedasticity-consistence robust inference based on HC3 standard errors was used. All models were tested by estimating the 95% confidence interval (CI) based on bootstrapping with 10,000 repetitions. To account for multiple comparisons across the three ROIs, we applied a Bonferroni correction to adjust the significance threshold to the moderation models. To avoid false positive results, we checked whether the CI did include 0. If this was not the case, we accepted the statistics as significant for *p* < 0.017. To assess whether the observed moderation effect was independent of PTSD-related variance, we conducted an additional analysis in which lifetime PTSD diagnosis (yes/no) was added as a covariate to the moderation model. This supplementary model was run on the subset of participants with complete PTSD data (*n* = 78), using the same PROCESS framework.

Since the sample size was fixed, we conducted post hoc power analyses using G*Power (version 3.1.9.7; Heinrich-Heine-Universität Düsseldorf, Germany) to assess the sensitivity of our findings. Power for the overall moderation models was calculated using the sample size (*n* = 81), a Bonferroni-corrected alpha level (*α* = 0.017), the full model *R*^2^, and an *F*-test with three predictors (CTQ, LPA, and CTQ × LPA).

Rather than just reporting the interaction term, significant moderation models were shown in detail with the simple slopes method for high (+1 SD above the mean), average (mean) and low (−1 SD below the mean) levels of the moderator (Min_A_) across the range of the focal variable (smaller volume to greater volume of ROI). This method allows for the visualization and interpretation of interaction effects, providing an intuitive and clear way to communicate the direction and strength of the interaction thereby facilitating a more straightforward interpretation of the relationship [43].

## Ethical considerations

The study was reviewed and approved by the Ethics board of the Medical Faculty Mannheim at Heidelberg University, Germany with protocol number 2018-616 *N*-MA, and was conducted in accordance with the Helsinki Declaration at the Central Institute of Mental Health in Mannheim. All participants provided their written (online) informed consent to participate in this study.

## Results

Our results showed that LPA average weekly minutes (Min_A_) did not appear to have a moderating effect on the volume of the hippocampus and ACC, as neither the overall models nor the interaction terms were statistically significant. Nonetheless, the model concerning the volume of the amygdala was significant and showed that Min_A_ moderated the relationship between CTQ and the volume of the amygdala. This model explained 8.1% of the variance in amygdala volume (*p* = 0.002) and the interaction of Min_A_ and CTQ contributed 7.9% of this variance, with a significant effect (*β* = −0.221 *p* ≤ 0.001). This indicates that the interaction between ACEs and amygdala volume was weakened by LPA average weekly minutes (Table 2). In a supplementary analysis controlling for lifetime PTSD diagnosis (*n* = 78), the ACE and LPA interaction on amygdala volume remained statistically significant. The pattern of results was consistent with the primary analysis (see Supplementary Table 1). Post hoc power analysis indicated that the overall moderation model for the amygdala had moderate power (0.75), in contrast, the hippocampus and ACC models were underpowered (Hippocampus = 0.21; ACC = 0.32).

**Table 2.** Moderation effect of LPA average weekly minutes on relationship between ACEs and ROI volume

	Hippocampus		Amygdala		ACC	
	ß	95% CI	ß	95% CI	ß	95% CI
CTQ	−0.076	(−0.282, 0.155)	0.082	(−0.132, 0.293)	0.033	(−0.200, 0.191)
Min_A_	−0.054	(−0.294, 0.374)	−0.027	(−0.215, 0.239)	−0.163	(−0.582, 0.039)
CTQ* Min_A_	−0.070	(−0.279, 0.241)	−0.221*	(−0.409, −0.059)	−0.153	(−0.462, 0.099)
*R*^2^	0.016		0.081		0.059	
*F_(3,77)_*(HC3)	3.241		5.362		0.558	
*p*	0.807		0.002		0.643	

*Note:* The table displays moderation analysis results. The *ß* values represent unstandardized regression coefficients. In the context of this moderation model, the *ß* coefficient for CTQ reflects the conditional effect of ACEs on brain volume when lifetime physical activity average weekly minutes (Min_A_) is zero; the coefficient for Min_A_ reflects its effect when CTQ is zero; and the interaction term (CTQ × Min_A_) represents the extent to which the association between ACEs and brain volume changes across different levels of LPA. Bootstrapped lower and upper 95% confidence intervals (CI) for each variable using 10.000 bootstrap samples. Bolded values indicate statistically significant interaction terms based on the Bonferroni-corrected threshold (*p* < 0.017).

**Figure 1.**
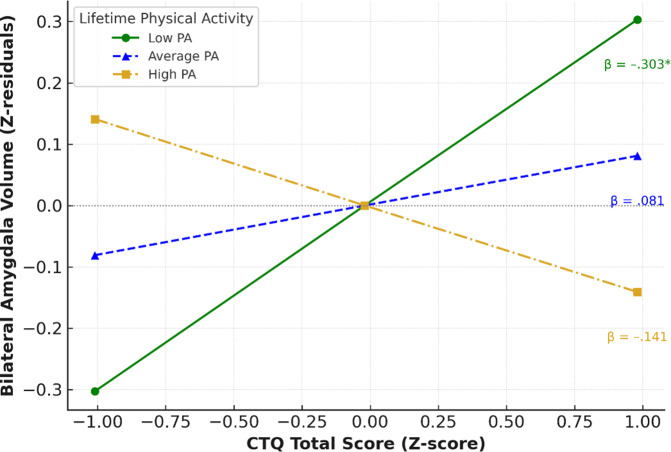
The moderation effect of lifetime physical activity average weekly minutes. *Note:* Simple slopes illustrating the interaction between ACEs (CTQ total score) and bilateral amygdala volume at low (−1 SD), average, and high (+1 SD) levels of lifetime physical activity. The x-axis shows Z-transformed CTQ scores; the y-axis shows standardized residuals of amygdala volume, adjusted for age and sex. The asterisk (*) indicates statistical significance at p < 0.05.

To get insights about the direction of interaction, the level of moderation effect analyzed and illustrated via the simple slope technique (Figure 1). In the moderation model, the simple slope of CTQ on amygdala volume was significant in the low LPA levels (one SD below the mean) (*β* = .303), but not in the high LPA levels (one SD above the mean) (*β* = −.141), indicating that the amygdala volume increased with increasing CTQ scores for those subjects with lower levels of LPA.

## Discussions

Our findings demonstrated that LPA had a moderating effect on amygdala volume in individuals with a history of ACEs. Specifically, in individuals with low, but not in those with high LPA average weekly minutes, ACEs were associated with increased volume of the amygdala. Although changes of the morphology of hippocampus and ACC were anticipated, LPA had no discernible impact on those regions. As no previous research has explored the relationship among these three variables, our results will be discussed in light of existing literature on brain morphological alterations in ACEs populations and the effect of PA on brain morphology.

The amygdala is an important brain structure and has attracted enormous interest of researchers due to its crucial role in salience detection, emotional memory, motor response, decision making and awareness. In studies on ACEs, the amygdala has been a primary focus as a key component of the central circuitry of emotion and stress sensitization [9]. Both animal and human studies have shown that ACEs influence the development of the amygdala, however the direction of this effect was not consistent in studies [20, 45]. In their meta-analysis, Paquala et al. (2016) investigated the alterations of gray matter regions of adults with ACEs history, including 38 original articles and findings on average a smaller amygdala [46]. However, Teicher et al. (2016) reported an enlargement of the amygdala following ACEs in their review [20]. In contrast to the majority of studies revealing amygdala alterations following ACEs, a recent meta-analysis of 39 data sets was not able to find any changes in amygdala volume [19]. Our findings add nuance to this literature by demonstrating that the association between ACEs and amygdala volume varies as a function of LPA levels (Table 2). Unlike many previous studies that employed correlational or main-effect models, our analysis tested an interaction effect using mean-centered variables. In this context, the coefficient for CTQ represents the effect of ACEs on amygdala volume when PA is at its mean, which does not correspond to a psychologically meaningful or uniform state in our sample. Therefore, we do not interpret the CTQ coefficient as a direct or general effect, but rather as part of a conditional framework in which the ACE–amygdala relationship depends on LPA levels. This distinction between simple correlation and moderation is critical: our findings do not suggest a uniform association between adversity and brain structure, but instead highlight the potential role modifiable, lifestyle-related behavioral components. Future longitudinal research is needed to clarify these dynamics over time, and to determine whether PA plays a causal, buffering effect on neurodevelopment following early life adversity.

The World Health Organization recently revealed that 31% of adults and 80% of adolescents engage in less PA than recommended [47]. Physical inactivity and sedentary lifestyle is known to be common among ACEs-exposed people since the first Kaiser–Permanente study, and supporting evidence is growing [48]. Although to a limited in number, studies have further shown that low PA exacerbates negative health outcomes of ACEs, including depressive symptoms, functional dependence, and poorer physical health [35]. While our study focused on the neuro-mechanistic effects of PA on ACEs-related brain volume changes, future research could explore how these PA- dependent changes impact physical and mental health. Chronic stress may drive amygdala volume alterations through dysregulation of the HPA axis [2, 3], and PA-moderated amygdala changes may, in turn, exacerbate HPA dysregulation, contributing to poor health outcomes. Beyond its potential relationship to mental and physical health, our findings of amygdala alterations, associated by low-level PA are concerning and indicate the necessity of implementing global PA promotion programs for adversity-affected communities.

The fact that we were unable to obtain any significant results from either the hippocampus or from the ACC came as a surprise. In the field of PA, the hippocampus is a region that has been extensively explored, highlighted as susceptible to transformation through PA. The majority of studies on ACEs have also revealed alterations in this region, not only for the structure but also the function and the connectivity of the hippocampus [10, 45]. Although the ACC is a relatively new area of study in the exercise field compared to the hippocampus, it has also garnered significant attention with studies demonstrating the benefits of exercise interventions, especially in people with mood disorders [11, 12]. The null findings in these regions should be interpreted cautiously, in particular as post hoc power analyses indicated that the hippocampus and ACC models were underpowered to detect small-to-moderate interaction effects. This limits our ability to draw definitive conclusions about the absence of PA-related modulation in these ROIs.

We did not examine intensity as a feature of PA, but this may be critical in understanding the region-specific effects observed. Emerging research suggests that different intensities of PA elicit distinct physiological responses, including variations in lactate, IGF-1, and BDNF levels, which may differentially impact brain regions involved in stress regulation and plasticity [49, 50]. It is possible that higher-intensity activity or more targeted forms of activity are required to induce measurable structural changes in the hippocampus or ACC, whereas the amygdala may be more broadly responsive to cumulative engagement regardless of intensity. Future studies should consider the role of intensity, as well as timing and consistency, in shaping how physical activity interacts with early adversity.

Although our study focused on three a priori ROIs based on well-established stress and resilience circuits, recent research suggests that ACE-related brain alterations may extend beyond the limbic system. Whole-brain and network-level analyses have begun to reveal broader cortical and subcortical involvement, including areas related to executive function, self-referential processing, and sensory integration [51, 52]. Future studies using multimodal neuroimaging and data-driven analytic approaches could complement our targeted ROI design and uncover additional neural pathways through which PA may buffer adversity. In addition, differentiating between specific ACE subtypes (e.g., abuse versus neglect) may reveal distinct vulnerability profiles and clarify the neural mechanisms most responsive to behavioral resilience factors like LPA.

There are some limitations that must be taken into account when considering the results of this work. While adopting a life-course approach to assess behavioral-resilience factors such as PA in the context of ACEs is critical, the cross-sectional and retrospective nature of this study presents inherent challenges. A prospective, longitudinal design would provide more robust insights into the interplay between PA, ACEs, and brain volume; however, such studies demand significant resources and necessitate initial evaluations of the connections among these three variables prior to allocating substantial scientific resources. As a practical initial step in this niche topic, we utilized self-reported PA, which is widely used in research. Yet, this approach is prone to recall bias, considering the fact that correlations between self-reported and objective measures of PA known to be generally low to moderate [53]. The following investigations might focus on integrating objective methodologies, including activity trackers, cardiorespiratory fitness testing, or longitudinal self-reports, to enhance measurement reliability.

Similarly, the retrospective assessment of ACEs using CTQ is also subject to self-report bias, particularly recall bias. While the observed low correlation between age and CTQ scores in our study may imply minimal bias, it may also reflect generational differences in ACEs exposure or advancements in prevention efforts over time. Importantly, despite the inherent limitations of retrospective self-report measures, previous research indicates that such assessments often underestimate-rather than exaggerate-the strength of associations between adversities and negative health outcomes, including alterations in brain structure [54-56]. This suggests that retrospective assessment may have led to a more conservative estimate of the true impact of ACEs in our study.

The generalizability of our findings is further limited by the relatively small and demographically imbalanced sample; particularly the predominance of female participants (83.3%) and the wide age range. Age and sex effects were statistically removed from ROI volumes prior to analysis, allowing us to account for variance attributable to these demographic factors. However, the design did not permit subgroup comparisons or exploration of sex-specific patterns in brain morphology or behavior. Given known sex and age related differences in stress sensitivity [13] brain structure [57], and PA engagement [58], future studies should aim for larger, sex-balanced and age-stratified samples to investigate these influences with sufficient statistical power. Finally, although age, sex, and PTSD diagnosis were accounted for statistically, the possibility of residual confounding from unmeasured factors, such as developmental timing of adversity, chronic stress exposure, or neurobiological heterogeneity, cannot be fully excluded.

Despite these limitations, this study is the first to investigate the moderating role of LPA on ACEs-related morphological brain changes. Our results suggest that PA behaviors have the potential to modulate the long-term effects of ACEs on brain structure. These findings underscore the importance of lifestyle factors in shaping ACEs-related outcomes and highlight the need for multidisciplinary approaches in research and clinical practice to effectively address the long-term impacts of childhood adversity on brain health.

## Supporting information

10.1192/j.eurpsy.2025.10116.sm001 Zehirlioglu et al. supplementary material Zehirlioglu et al. supplementary material

## Data Availability

The datasets including extracted ROI volumes analyzed during the current study are publicly available in the OSF repository https://osf.io/cj9va/files/osfstorage. Anonymized raw neuroimaging data are available upon reasonable request from the corresponding authors, subject to local ethics committee requirements. The standard preprocessing script is used and can be found here https://neuro-jena.github.io/enigma-cat12/#standalone. The code of moderation analysis is publicly available in the OSF repository https://osf.io/cj9va/files/osfstorage.

## Supplementary material

The supplementary material for this article can be found at http://doi.org/10.1192/j.eurpsy.2025.10116.
