# Supplementary material for: The moderating effect of lifetime physical activity on brain alterations related to adverse childhood experiences
Source: Eur Psychiatry. 2025 Oct 20;68(1):e168. doi: 10.1192/j.eurpsy.2025.10116 (PMC12646122; doi:10.1192/j.eurpsy.2025.10116)
Supplement: Zehirlioglu et al. supplementary material [file S0924933825101168sup001.docx]

**Post Hoc Analyses**

To evaluate the robustness of our findings, we conducted additional moderation analyses including a binary indicator of history of lifetime psychopathology based on The Structured Clinical Interview for DSM-5 (Table 1). The significant moderation effects remained robust, underscoring the independent contribution of LPA in moderating the impact of ACE on amygdala volume.

The CTQ × MinA interaction again remained significant for amygdala volume (β = -0.208, p < .0001). These results further support the interpretation that the observed moderation effect is not confounded by lifetime diagnostic history.

**Table 2. Moderation Effect of LPA Average Weekly Minutes on Relationship between ACEs and ROI Volume Controlling for History of PTSD**

|  | Hippocampus | | Amygdala | | ACC | |
| --- | --- | --- | --- | --- | --- | --- |
|  | ß | 95% CI | ß | 95% CI | ß | 95% CI |
| CTQ | -0.002 | (-0.275, 0.271) | 0.164 | (-0.128, 0.456) | 0.033 | (-0.280, 0.347) |
| Min_A_ | -0.064 | (-0.370, 0.241) | -0.027 | (-0.207, 0.153) | -0.154 | (-0.556, 0.247) |
| CTQ^*^ Min_A_ | -0.058 | (-0.278, 0.161) | **-0.208*** | (-0.313, -0.102) | -0.155 | (-0.505, 0.195) |
| Lifetime Diagnosis of PTSD | -0.288 | (-0.848, 0.272) | 0.0360 | (-0.808, 0.298) | 0.101 | (-0.499, 0.703) |
| R^2^  F*_(4,73)_*(HC3)  p | 0.030  0.410  0.801 | | 0.090  4.469  0.003 | | 0.061  0.523  0.719 | |

Moderation analysis results. B values represent unstandardized regression coefficients. In the context of this moderation model, the B coefficient for CTQ reflects the conditional effect of ACEs on brain volume when physical activity (PA) is zero; the coefficient for PA reflects its effect when CTQ is zero; and the interaction term (CTQ × PA) represents the extent to which the association between ACEs and brain volume changes across different levels of PA. 95% confidence intervals were obtained using 10,000 bootstrap samples. Bolded values indicate statistically significant interaction terms based on the Bonferroni-corrected threshold (p < 0.017).
